# Study protocol: understanding the pathophysiologic mechanisms underlying delirium in older people undergoing hip fracture surgery

**DOI:** 10.1186/s12877-021-02584-1

**Published:** 2021-11-04

**Authors:** R. Gamberale, C. D’Orlando, S. Brunelli, R. Meneveri, P. Mazzola, G. Foti, G. Bellani, G. Zatti, D. Munegato, S. Volpato, A. Zurlo, G. Caruso, A. Andreano, M. G. Valsecchi, G. Bellelli

**Affiliations:** 1 School of Medicine and Surgery, University of Milano-Bicocca, Milan, Italy; 2 Acute Geriatric Unit, San Gerardo Hospital, Monza, Italy; 3 Orthogeriatric Unit, Arcispedale S. Anna, University of Ferrara, Ferrara, Italy; 4 Department of Medical Sciences, University of Ferrara, Ferrara, Italy; 5 Department of Biomedical and Surgical Specialist Sciences, University of Ferrara, Ferrara, Italy

**Keywords:** Postoperative delirium, Neuroinflammation, Sarcopenia, Biomarkers

## Abstract

**Background:**

Postoperative delirium (POD) is a common complication of older people undergoing hip fracture surgery, which negatively affects clinical- and healthcare-related outcomes. Unfortunately, POD pathophysiology is still largely unknown, despite previous studies showing that neuroinflammation, neuroendocrine dysfunction, increased reactive oxidative stress (ROS), and endothelial dysfunctions may be involved. There is also evidence that many of the pathophysiological mechanisms which are involved in delirium are involved in sarcopenia too.

This article describes the protocol of a pilot study to evaluate the feasibility of a larger one that will explore the pathophysiological mechanisms correlating POD with sarcopenia. We will analyse whether various biomarkers reflecting neuroinflammation, ROS, neuroendocrine disorders, and microvasculature lesions will be simultaneously expressed in in the blood, cerebrospinal fluid (CSF), and muscles of patients developing POD.

**Methods:**

Two centres will be involved in this study, each recruiting a convenient sample of ten older patients with hip fracture. All of them will undergo a baseline Comprehensive Geriatric Assessment, which will be used to construct a Rockwood-based Frailty Index (FI). Blood samples will be collected for each patient on the day of surgery and 1 day before. Additionally, CSF and muscle fragments will be taken and given to a biologist for subsequent analyses. The presence of POD will be assessed in each patient every morning until hospital discharge using the 4AT. Delirium subtypes and severity will be assessed using the Delirium Motor Subtype Scale-4 and the Delirium-O-Meter, respectively. We will also evaluate the patient’s functional status at discharge, using the Cumulated Ambulation Score.

**Discussion:**

This study will be the first to correlate biomarkers of blood, CSF, and muscle in older patients with hip fracture.

**Supplementary Information:**

The online version contains supplementary material available at 10.1186/s12877-021-02584-1.

## Background

Delirium is a neuropsychiatric disorder characterized by impairment of many cognitive functions (mainly attention and executive functions) that develops as the result of one or more acute clinical problems or because of drugs or due to mixed aetiology, It tends to fluctuate, with patient’s cognition and awareness rapidly changing from normal to either hyperactive or hypoactive states [1].

The term postoperative delirium (POD) refers to a condition of delirium that develops after surgery. Up to 40% of older people are affected by POD after hip fracture surgery [2, 3]. It is a life-threatening condition, with the risk of death at 6 months being particularly elevated for patients presenting with the hypokinetic or mixed POD subtypes [3, 4].

The pathophysiology of POD remains largely unknown. However, an increasing body of evidence suggests that neuroinflammation and a damage of blood-brain barrier (BBB) are involved: endothelium lesions at the BBB level can result in an increased permeability, allowing immune cells, cytokines and other neuroinflammatory products to enter brain parenchyma. This mechanism is particularly likely to occur in old and demented subjects with hip fracture, as elevated serum levels of pro-inflammatory cytokines including tumour necrosis factor-α (TNF-α), interleukin-1 (IL-1), IL-6, and IL-8 have been reported in these patients [5]. After crossing BBB, inflammatory mediators can activate microglia, leading to synaptic and neuronal dysfunction, and, eventually, delirium [6, 7].

Inflammatory mediators promote the infiltration of myeloid immune system cells in the central nervous system, such as peripheral blood mononuclear cells (PBMCs), which have been reported to have a role in neuroinflammation in mouse models of Alzheimer’s disease [8]. Animal models have also shown that systemic inflammation accelerates the existing neuropathology, such as by microglial priming in acute cognitive and motor deficits in ME7 models of prion disease [9].

A second pathophysiological mechanism has been proposed to explain delirium, relying on the oxidative stress hypothesis [7]. Under specific circumstances, such as during surgery, the brain is highly vulnerable to damage of oxidative stress, possibly leading to neuronal dysfunction [10]. Neopterin is a pteridine synthesized by monocytes, macrophages, microglia, and dendritic cells from guanosine triphosphate, which can amplify the cytotoxicity of reactive oxygen species (ROS) from the macrophage oxidative burst, thereby increasing the cytocidal effects of the cell-mediated immune response [11]. Neopterin is being studied in many fields as a biomarker for both cell-mediated immunity and oxidative stress [11, 12]. Previous studies have shown that neopterin levels become more elevated in patients with POD than in controls, both in the plasma and in the cerebrospinal fluid (CSF) [12, 13].

A third pathophysiological hypothesis proposes that delirium represents a response to acute or chronic stress, mediated by abnormally high glucocorticoid (GC) levels [7]. GCs exert widespread actions in the Central Nervous System, including the regulation of gene transcription, cellular signalling, modulation of synaptic structure, regulation of glial functions and effects on behaviour [14].

Previous studies have shown that patients who experience POD have higher postoperative cortisol levels with enhanced postoperative elevation in relation to baseline [15]. Over time, repeated or prolonged high GC levels impair the ability of neurons to survive after various metabolic insults, leading to a general vulnerability in brain neurons, also known as the “aberrant stress response” [16, 17].

All these hypotheses should not be considered as mutually exclusive pathophysiological mechanisms for delirium occurrence but, on the contrary, they should be regarded as complementary, with many areas of intersection and reciprocal influence [7].

Sarcopenia is defined as a condition of reduced skeletal muscle mass and strength, which is associated with several negative outcomes [18]. It is a common condition in older patients with hip fracture, ranging from 21 to 74% in men and 12 to 68% in women, depending on the definition used [19, 20]. It has a complex multifactorial pathogenesis involving not only age-related changes in neuromuscular function, muscle protein turnover, and hormone levels but also a chronic pro-inflammatory state and oxidative stress [21]. In recent years, there has been a blossoming of studies showing that dysregulation of inflammatory mediators, ROS production, endocrine systems and microvascular lesions play a key role in sarcopenia pathophysiology [22, 23]. This evidence globally suggests that delirium and sarcopenia might share similar pathophysiologic mechanisms and a potential biological cross-talk. Evidence of a brain-muscle cross-talk is also supported by other studies showing a positive correlation between sarcopenia, cognitive impairment, and slow gait speed [24, 25]. However, there are no studies in patients with hip fracture that have explored the existence of shared pathophysiological mechanisms linking brain and muscle disorders. Thus, the current project aims to address this gap. This article describes the protocol of a pilot study to evaluate the feasibility of a larger study that will explore the pathophysiological mechanisms correlating POD with sarcopenia. We will investigate if selected biomarkers reflecting neuroinflammation, ROS, neuroendocrine disorders, and microvasculature lesions will be simultaneously expressed in the blood, cerebrospinal fluid, and skeletal muscle tissue of patients who developed POD after hip fracture surgery. The potential implications of the findings of such study will be various, encompassing both the field of delirium and sarcopenia.

## Methods/design

### Study design

This is a feasibility study for the prospective collection of blood, CSF, and muscular samples in older patients undergoing surgery for hip fracture. The study will be conducted at the Orthogeriatric Units (OGUs) of the San Gerardo hospital, Monza and of the Azienda Ospedaliero-Universitaria S. Anna, Ferrara, Italy from October 2021 to October 2022.

### Patient recruitment

Ten patients per centre will be recruited. All patients admitted to the participating orthogeriatric units (OGUs) will be screened for participation in the study, after their arrival to the hospital and before surgery. Each patient will be asked to give informed consent for the participation in the study; if he/she is able to give the consent because of severe cognitive impairment, the consent will be asked to patients’ next of kin.

### Inclusion and exclusion criteria

Inclusion criteria for this study will be: age 65 years and above; a femoral neck fracture (FNF) caused by a low energy trauma (defined as a fall from less than 1 m) needing surgical repair; suitability for subarachnoid anaesthesia; willingness to participate in the study and ability to understand and speak Italian.

Exclusion criteria will be: a pathologic FNF; ongoing anti-inflammatory or corticosteroid treatment; aphasia, advanced Parkinson’s disease or Parkinsonism (i.e., a score > 4 at Hoehn and Yahr Scale) [26]; advanced dementia (i.e., a score > 6 at Global Deterioration Scale) [27] and; ascertainment of pre-surgical delirium. Delirium will be evaluated with the 4AT test [28]. The 4AT is a brief and pragmatic tool for the screening of delirium which has demonstrated acceptable diagnostic test accuracy in acute medical wards [28, 29]. A score higher than 4 is strongly predictive of delirium. Patients who screen positive during the 4AT assessment will be further evaluated for the presence of delirium using the Diagnostic and Statistical Manual of Mental Disorders (DSM-5), fifth Edition criteria [1]. If delirium is confirmed according to DSM-5 criteria, the patient will be excluded from the study.

### Assessment and data collection

Table 1 reports the Gantt charts of the time and the specialist who will carry on the procedures of the study.

**Table 1:**
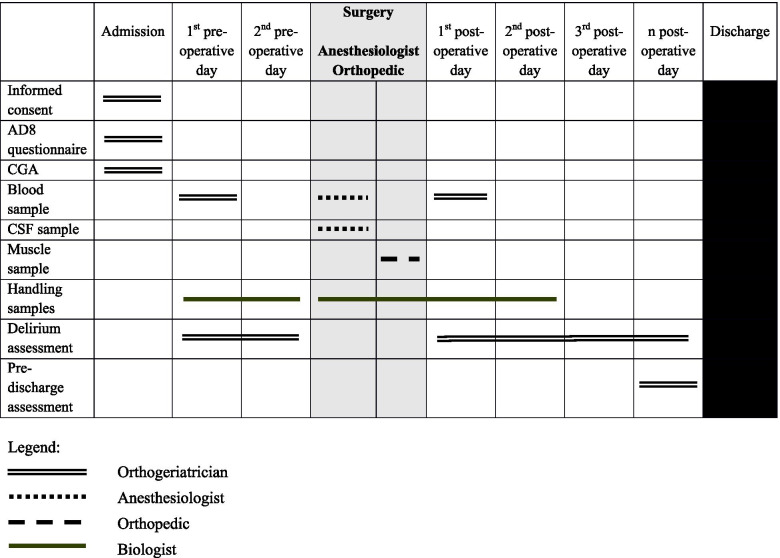
Gantt chart of times and participants

All eligible patients will undergo a Comprehensive Geriatric Assessment (CGA), a globally approved method used to evaluate the older patients [30]. Information on demographic characteristics and clinical, functional, and nutritional data will be collected on admission by a geriatrician at each centre, using a set of validated tools as previously described [31]. This includes the Katz’ Activity of Daily Living (ADL) [32], the New Mobility Score (NMS) [33], and the Mini Nutritional Assessment Short-Form (MNA-SF) [34]. Sarcopenia will be defined according to European Working Group on Sarcopenia in Older People (EWGSOP2) criteria [18]. Muscle strength will be assessed by grip strength, measured using a hand-held dynamometer with the patient lying in bed with a 30° flexed back, with the shoulder abducted and rotated in a neutral position, the elbow resting on the bed and the wrist in a neutral position [35]. Three trials for each hand will be performed, and the highest value of the strongest hand will be used in the analyses. Appendicular skeletal muscle (ASM) mass will be estimated, according to the equations validated by Santos in 2019 [36]. In accordance with the EWGSOP2 criteria and cut-off points, sarcopenia will be defined as the presence of both low muscle strength and low muscle mass. Comorbidity will be assessed using the Charlson Comorbidity Index (CCI) and information on drugs use will be obtained by reviewing the patient’s medical records [37]. Other data to be collected on admission will include the assessment of both visual and hearing impairment. Cognitive impairment will be screened according to the score reported on the AD8 questionnaire [38]. The AD8 is not a tool to define dementia but it can serve to detect early cognitive changes associated with dementia-related illnesses. In this study, the AD8 will be administered to an informant since the patients we are planning to recruit almost certainly will not be suitable for cognitive screens due to their acute state. The AD8 contains 8 items that test for memory, orientation, judgment, and function. Cut points are normal cognition 0–1; impairment in cognition 2 or greater. Using the above-mentioned variables, a Frailty Index (FI) will be calculated on admission. The FI is based on the Rockwood’s theoretical concept that frailty derives from the age-related accumulation of deficits [39]. It considers clinical signs, symptoms, diseases, disabilities, psychosocial risk factors, and geriatric syndromes, resulting in a score that has shown to be strongly associated with negative health-related outcomes (including hospitalizations, institutionalization, and mortality) [39]. Each deficit included in the FI will be coded as 0, 0.5, or 1 to indicate its absence, partial presence, or presence, respectively. Overall, at least 40 variables will be included in the computation of the FI, which will make our variable relatively robust [39]. Each participant’s FI will be calculated as the ratio between the presented deficits divided by the number of evaluated items. Participants with a FI score 0.25 will be considered frail, whereas those with lower values will be considered non-frail [40]. Additionally, a further measure of frailty will be obtained using the Canadian Frailty Scale [41].

Before surgery, the anaesthesiologist will assess the ASA score, a commonly used tool to evaluate a patient’s risk of dying, and the Sequential Organ Failure Assessment (SOFA) score [42]. On the day of surgery, type of FNF, type of anaesthesia and duration, time-from-fracture-to-surgery, and type of surgery will also be recorded.

After surgery, each patient will be evaluated every morning until hospital discharge to assess the development of POD. This assessment will include the same procedures as for evaluating the presence of pre-surgical delirium, according to a previous protocol [31]. This information will be used to support or reject the diagnosis of delirium. Additionally, patients with a confirmed diagnosis of delirium will undergo the Delirium Motor Subtype Scale-4 (DMSS) to categorize delirium into motor subtypes (i.e., hyperactive, hypoactive, mixed, and non- hyperactive-non-hypoactive), and the Delirium-O-Meter (D-O-M), to assess delirium severity [43, 44]. Both these tools are not under license and are free to use. Finally, postoperative pain will be evaluated with the Verbal Numeric Pain Scale in mentally healthy subjects [45], while the Pain Assessment in Advanced Dementia (PAINAD) will be used for patients with severe cognitive impairment [46].

Before discharge, change in haemoglobin serum levels from admission to discharge and the number of units of blood transfused will be recorded. At discharge we will assess the patient’s calf circumference, the grip strength with the hand dynamometer, and the functional status with the Cumulated Ambulation Score (CAS) [47]. The CAS describes the patient’s independence in three activities (getting in and out of bed, sit-to-stand-to-sit from a chair, and walking). Each activity is assessed on a three-point ordinal scale from 0 to 2 (0 = Unable to perform the task despite human assistance and verbal cueing, 1 = Able to perform the task with human assistance and/or verbal cueing from one or more persons, 2 = Able to safely, without human assistance or verbal cueing, use of a walking aid allowed) resulting in a total daily CAS score ranging from zero to six. Finally, the length of hospital stay and the destination at discharge (i.e., home, rehabilitation facility, or nursing home) will be recorded.

### Sample collection, sample handling and processing, and laboratory procedures

All patients giving informed consent to the study will be eligible for the collection of blood, CSF, and muscle samples. Patients who die before or during surgical procedure will be considered drop-outs and therefore their samples will not be analysed.

The first blood sample (4–5 mL obtained by venous puncture) will be collected on the day after OGU admission. On the day of surgery, CSF will be collected during subarachnoid anaesthesia. After dura mater puncture, 1.5 mL of CSF will be collected in sterile conditions, before injection of the local anaesthetic. Thus, the patient will not undergo any additional invasive procedure. Another sample of blood will be collected by venous puncture right after CSF collection (4–5 mL per patient). Polypropylene tubes with EDTA (to avoid protein aggregation on the internal surface of the tube) will be used for the collection of blood and CSF.

Muscle tissue will be collected by local biopsy during surgery, once the patient is anaesthetized, so that no pain or damage will be caused. A compact longitudinal cylinder (optimal size: 1 cm length, 0.5 cm thickness) will be taken with a biopsy needle from the vastus lateralis muscle, carefully keeping fibres in a longitudinal orientation. The sample will be inserted in a polypropylene tube containing RNA Later Solution (Sigma-Aldrich), which allows to preserve sample and RNA integrity. All samples will be delivered to the researcher of reference as soon as possible.

The procedure will be as follows: the patient will be admitted in the operating room carrying a sealable bag containing the tubes for the collection of blood, CSF, and the muscle samples. Inside the bag, written information to remind anaesthesiologists and surgeons about the procedures will be reported. Finally, another blood sample will be collected the day after surgery.

Once the collection is complete, all samples will be given to a biologist. Blood and CSF will be centrifuged at 2000 x g for 10 min; centrifuged CSF will be aliquoted in cryotubes (0.5–1.0 ml per cryotube), while plasma will be collected from blood after centrifugation and will be in turn aliquoted in cryotubes (0.5–1.0 ml per cryotube). All the samples will be stored at − 80 °C in dedicated freezers. The list of the biomarkers which we have planned to assess are shown in Table 2, along with the reference of previous studies supporting the rationale (Supplementary material). All of them will be measured using multiplex ELISA assays (R&D Systems) in plasma and CSF, while biomarkers in PBMCs and muscles will be measured by RT-qPCR. RNA will be isolated from PBMCs and muscle cells using specific extraction kits (Qiagen) and miRNA will be isolated from exosomes purified from plasma using exoQuick (SBI) [48].

**Table 2:** Selected biomarkers for the study

Biomarkers	Blood (plasma)	Blood (PBMCs)	Cerebrospinal fluid	Muscle
**Pro-inflammatory cytokines**	IL-6 [S1, S2, S3] IL-8 [S1, S2, S3] IL-1β [S1, S2, S3] TNF-α [S1, S2, S3] IFNγ [S1, S2, S3]	IL-6 [S1, S2, S3] IL-8 [S1, S2, S3] IL-1β [S1, S2, S3] TNF-α [S1, S2, S3] IFNγ [S1, S2, S3]	IL-6 [S1, S2, S3] IL-8 [S1, S2, S3] IL-1β [S1, S2, S3] TNF-α [S1, S2, S3] IFNγ [S1, S2, S3]	IL-6 [S1, S2, S3] IL-8 [S1, S2, S3] IL-1β [S1, S2, S3] TNF-α [S1, S2, S3] IFNγ [S1, S2, S3]
**Anti-inflammatory cytokines**	IL-10 [S1, S2, S3] IL-37 [S4] TGF-β [S3]	IL-10 [S1, S2, S3] IL-37 [S4] TGF-β [S3]	IL-10 [S1, S2, S3] IL-37 [S4] TGF-β [S3]	IL-10 [S1, S2, S3] IL-37 [S4] TGF-β [S3]
**Muscular damage and regeneration**				Atrogin-1 [S5] MuRF-1 [S5] Myostatin [S5] Follistatin [S5] Activin [S5] Activin receptor type II [S5] Myosin heavy chain type II [S5] Myosin heavy chain type VII [S5]
**Muscle differentiation**				PAX7 [S5] MyoD [S5] Myogenin [S5] MYF5 [S5] MEF2A [S5]
**Endothelial damage**	CD-31 [S6] S-100β [S3, S7] Von Willebrand factor [S6]	CD-31 [S6] S-100β [S3, S7] Von Willebrand factor [S6]	CD-31 [S6] S-100β [S3, S7] Von Willebrand factor [S6]	
**Transport proteins**	Albumin [S1, S2]	Albumin [S1, S2]	Albumin [S1, S2]	
**Immune system activation**	Neopterin [S8]	Neopterin [S8]	Neopterin [S8]	
**Neurodegeneration**	Neurofilament light chain [S9]	Neurofilament light chain [S9]	Neurofilament light chain [S9]	
**Stress response**	Cortisol [S10, S11]		Cortisol [S10, S11]	

Biomarkers coming from fluids (plasma and CSF) will be measured by protein quantification assays, while biomarkers coming from cells (PBMCs and myocytes) will be measured by mRNA expression analysis

Part of the muscle will be embedded, stored at − 80 °C and cut at the cryostat. We will perform immunohistochemistry and immunofluorescence techniques to evaluate muscle composition in terms of proportion of type I and type II fibers and the degree of intra- and intermuscular adipose tissue. This analysis will be done by the laboratory directed by two coauthors (RG and CD).

### Outcome measures

The primary outcome measure of the pilot study will be the ability to collect the pre-planned amount of blood, CSF, and muscle samples in the centres participating in the study. We will measure the number of biological samples collected, the number of those suitable for further processing, and the number of analyses performed. Finally, the reasons for failure in the collection or storage of the biological samples will be considered. Additionally, we will compare the characteristics of patients developing POD in terms of clinical features and difference in blood, CSF, and muscle biomarkers. Overall, we plan to screen no more than 20 patients per centre to achieve the expected sample size. Every centre admits an average number of 300 hip fractured patients per year.

### Statistical analysis and outcome measures

The data will be analysed at the Biostatistics Centre for Clinical Epidemiology of the Milano-Bicocca University. The study variables will be described as mean, standard deviation or median and interquartile range, if continuous, or as frequencies and percentages if categorical. The characteristics of patients who will develop POD or not will be compared using the chi-squared or Fisher test for the categorical variables, and with the t-test for mean comparison or the Mann-Whitney U test for the continuous ones. The number of patients needed to detect a significant difference for some specific biomarkers (inflammatory, ROS, and cortisol) in the blood and CSF of hip fractured patients was calculated. However, we do not know whether this expected sample size would be informative to detect a significant difference with the same biomarkers in the muscle too. Therefore, we opted for a convenience sample. The biomarker levels in all the biological samples (blood, CSF, and muscle) will be used to estimate the difference and variability, which will constitute the premises to conduct a larger multicentre study.

### Ethics and dissemination

The study has been approved by the Brianza Ethics Committee and will be conducted in accordance with the European Union (EU) 2016/679 and 2016/680 directive and guidelines. Information will be collected on the Research Electronic Data Capture platform [49]. Site access will be protected by a password, to ensure data safety and confidentiality. Passwords will be given to the researchers by a data manager of the Milano-Bicocca University. Every patient will be given a univocal alphanumeric code, to which all the data collected during the study will be assigned. The code will be copied and stored on an encrypted file. Data will be anonymized to avoid patients’ recognition (standard anonymization ISO 25237:2017).

## Discussion

Delirium, and in particular POD, is common and associated with several negative outcomes. However, its pathophysiology is still largely unknown, which is an important barrier to the identification of patients at risk, as well as of those who may respond to specific treatments.

Previous studies have tried to assess the mechanisms potentially involved in delirium pathophysiology, by measuring biomarkers of inflammation, neuroendocrine dysfunction, increased oxidative stress at the blood and CSF levels. However, most of these studies were carried out by separately assessing the putative biomarkers either in the blood or CSF, whereas only a minority assessed these biomarkers simultaneously in the blood and CSF.

The most studied markers are the ones related to the inflammatory response since the well-known relationship between systemic and brain inflammation. A recent systematic review has shown that IL-1β, IL-6, IL-8, IL-10, and IFN-γ are significantly associated with delirium onset, displaying higher levels, both in plasma and CSF, in relation to baseline [50]. Not only cytokines, but also cortisol has been investigated in the context of POD, showing how the stress response to surgery may lead to an increase of its serum and plasmatic level [51]. Additionally, those patients at high risk for delirium (eg, older adults with baseline cognitive impairment) exhibit sustained high cortisol levels after major stressors likely due to impaired feedback regulation of the hypothalamic-pituitary-adrenal axis [52]. This feedback regulation could be a factor driving towards delirium [52]. There is also evidence that altered oxidative metabolism may be one of the initiating causes of delirium development. In effect, disordered oxidative response can cause abnormal neurotransmitter synthesis, metabolism, and release, as well as free radical production and failure to effectively remove neurotoxic by-products [53]. It may also be hypothesized that neuroinflammation causes BBB permeability disruption, as suspected by increased levels of S100β (a calcium-binding protein with cytokine-like properties) and changes in synaptic transmission, neural excitability, and cerebral blood flow, leading to delirium [53].

Given that many of the described pathophysiologic mechanisms of delirium have been reported to be potentially involved in sarcopenia, it appears crucial to put in practice a multicentre study aimed at exploring the existence of shared biomarkers between the two. Furthermore, focusing on the biomarker’s kinetics rather than on single measurements, would be helpful in determining their potential role in the pathogenesis of POD. However, no study has assessed the potential cross-talk and interaction between the blood and CSF districts and the muscle of hip fracture patients, despite preliminary evidence reporting that delirium is associated with sarcopenia [54]. This study may, thus, be the first aiming at assessing this potential triple interaction. Furthermore, because sarcopenia is known to be a major determinant of frailty [55], which in turn is related to delirium [56], this study may help to discover potential pathophysiological mechanisms of frailty too. Our goal is to create the premise to fill the existing gaps in this field. Should we find molecular patterns activated synchronically in different frameworks, there would be several important outcomes for the research, allowing a swift recognition of patients at risk of developing POD in time and a better prognosis. This is a feasibility study to assess the capability of two multidisciplinary teams, focusing on the same project but located in two different hospitals, in collecting, storing, and analysing the samples obtained from the blood, CSF, and muscle of hip fracture patients. If this study demonstrates that the two hospital’s teams will be able to achieve this goal, we will proceed with a further multicentre study, which will be powered accordingly.

In conclusion, this pilot study aims to pave the way for a larger multicentre study which will explore the in-depth pathophysiological mechanisms linking POD development and muscle abnormalities. It will also potentially inform future studies on POD, as well as on sarcopenia, aiming at early identification of patients at risk for the two conditions.

## Supplementary Information

**Additional file 1.**

## Data Availability

A de-identified dataset may be made available upon reasonable request of the corresponding author once the study is completed.

## Abbreviations

POD: Postoperative delirium
BBB: Blood brain barrier
ROS: Reactive oxygen species
CSF: Cerebrospinal fluid.
GC: Glucocorticoid
OGUs: Orthogeriatric units
FNF: Femoral neck fracture
DSM-5: Diagnostic and Statistical Manual of Mental Disorders
CGA: Comprehensive Geriatric Assessment
ADL: Activity of Daily Living
NMS: New Mobility Score
MNA-SF: Mini Nutritional Assessment Short-Form
CCI: Charlson Comorbidity Index
ASM: Appendicular skeletal muscle mass
FI: Frailty Index
SOFA: Sequential Organ Failure Assessment
DMSS: Delirium Motor Subtype Scale-4
D-O-M: Delirium-O-Meter
PAINAD: Pain Assessment in Advanced Dementia
CAS: Cumulated Ambulation Score
PBMCs: Peripheral Blood Mononuclear Cells
IL: Interleukin
TNF-α: Tumor necrosis factor alpha
IFN-γ: Interferon gamma
