# Supplementary material for: Study protocol: understanding the pathophysiologic mechanisms underlying delirium in older people undergoing hip fracture surgery
Source: BMC Geriatr. 2021 Nov 4;21:633. doi: 10.1186/s12877-021-02584-1 (PMC8567587; doi:10.1186/s12877-021-02584-1)
Supplement: Supplementary file 1 — Additional file 1. [file 12877_2021_2584_MOESM1_ESM.docx]

**Supplementary material**

**References Table 2:**

1. Bromander S, Anckarsäter R, Kristiansson M, et al. Changes in serum and cerebrospinal fluid cytokines in response to non-neurological surgery: an observational study. *J Neuroinflammation* 2012;9:242.
2. Hall RJ, Watne LO, Cunningham E, et al. CSF Biomarkers in Delirium: A Systematic Review. *Int J Geriatr Psychiatry* 2018; 33 (11), 1479-1500.
3. Cerejeira J, Firmino H, Vaz-Serra A, et al. The neuroinflammatory hypothesis of delirium. *Acta Neuropathologica* 2010; 119 (6), 737–754.
4. Androsova G, Krause R, Winterer G et al. Biomarkers of postoperative delirium and cognitive dysfunction. *Front. Aging Neurosci* 2015;7:112.
5. D'Orlando C, Marzetti E, François S, et al. Gastric Cancer Does Not Affect the Expression of Atrophy-Related Genes in Human Skeletal Muscle. *Muscle Nerve* 2014;49(4),528-33.
6. Goncharov NV, Nadeev AD, Jenkins RO, et al. Markers and Biomarkers of Endothelium: When Something Is Rotten in the State. *Oxid Med Cell Longev* 2017;2017:9759735.
7. Erikson K, Ala-Kokko TI, Koskenkari J, et al. Elevated Serum S-100β in Patients With Septic Shock Is Associated With Delirium. *Acta Anaesthesiol Scand* 2019;63(1):69-73.
8. Hall RJ, Watne LO, Idland AV, et al. Cerebrospinal fluid levels of neopterin are elevated in delirium after hip fracture. *J Neuroinflammation* 2016;13:170.
9. Casey CP, Lindroth H, Mohanty R, et al. Postoperative delirium is associated with increased plasma neurofilament light. *Brain* 2020;143(1),47–54.
10. Pearson A, de Vries A, Middleton SD, et al. Cerebrospinal fluid cortisol levels are higher in patients with delirium versus controls. *BMC Res Notes* 2010;3:33.
11. Kazmierski J, Banys A, Latek J, et al. Cortisol levels and neuropsychiatric diagnosis as markers of postoperative delirium: a prospective cohort study. *Crit Care* 2013;17:R38.
